# Supplementary material for: Nucleosome reorganisation in breast cancer tissues
Source: Clin Epigenetics. 2024 Apr 1;16:50. doi: 10.1186/s13148-024-01656-4 (PMC10986098; doi:10.1186/s13148-024-01656-4)
Supplement: Supplementary file 1 — Additional file 1: Supplementary Materials. [file 13148_2024_1656_MOESM1_ESM.pdf]

Supplementary Materials

Nucleosome reorganisation in breast cancer tissues

Divya R. Jacob[1,^], Wilfried M. Guiblet[2,^], Hulkar Mamayusupova[1,^], Mariya Shtumpf[1], Isabella Ciuta[1], Luminita Ruje[1], Svetlana Gretton[1,3], Milena Bikova[1], Clark Correa[1], Emily Dellow[1], Shivam P. Agrawal[1], Navid Shafiei[1], Anastasija Drobysevskaja [1], Chris M. Armstrong[1], Jonathan D. G. Lam[1], Yevhen Vainshtein[4], Christopher T. Clarkson[1,5], Graeme J. Thorn[1,6], Kai Sohn[4], Madapura M. Pradeepa[1,7], Sankaran Chandrasekharan[8], Greg N. Brooke[1], Elena Klenova[1], Victor B. Zhurkin[2,*] and Vladimir B. Teif[1,*]

[1] School of Life Sciences, University of Essex, Wivenhoe Park, Colchester, CO4 3SQ, UK

[2] Center for Cancer Research, National Cancer Institute, National Institutes of Health, Bethesda, MD 20892, USA

[3] Current address: School of (EAST) Engineering, Arts, Science and Technology, University of Suffolk, James Hehir Building, University Avenue, Ipswich, Suffolk, IP3 0FS, UK

[4] Fraunhofer-Institut für Grenzflächen- und Bioverfahrenstechnik IGB, Nobelstraße 12, Stuttgart, 70569, Germany.

[5] Current address: University College London, Gower St, Bloomsbury, London WC1E 6BT, UK.

[6] Current address: Barts Cancer Institute, Queen Mary University of London, Charterhouse Square, London, EC1M 6BQ, UK.

[7] Current address: Blizard Institute, Barts and the London School of Medicine and Dentistry, Queen Mary University of London, London, E1 2AT, UK

[8] Colchester General Hospital, East Suffolk and North Essex NHS Foundation Trust, Turner Road, Colchester, CO4 5JL, UK

^ These authors contributed equally

* Correspondence should be addressed to Vladimir B Teif (vteif@essex.ac.uk) or Victor B Zhurkin (zhurkin@nih.gov)

Further information and requests for resources should be directed to and will be fulfilled by the lead contact, Vladimir B Teif (vteif@essex.ac.uk).

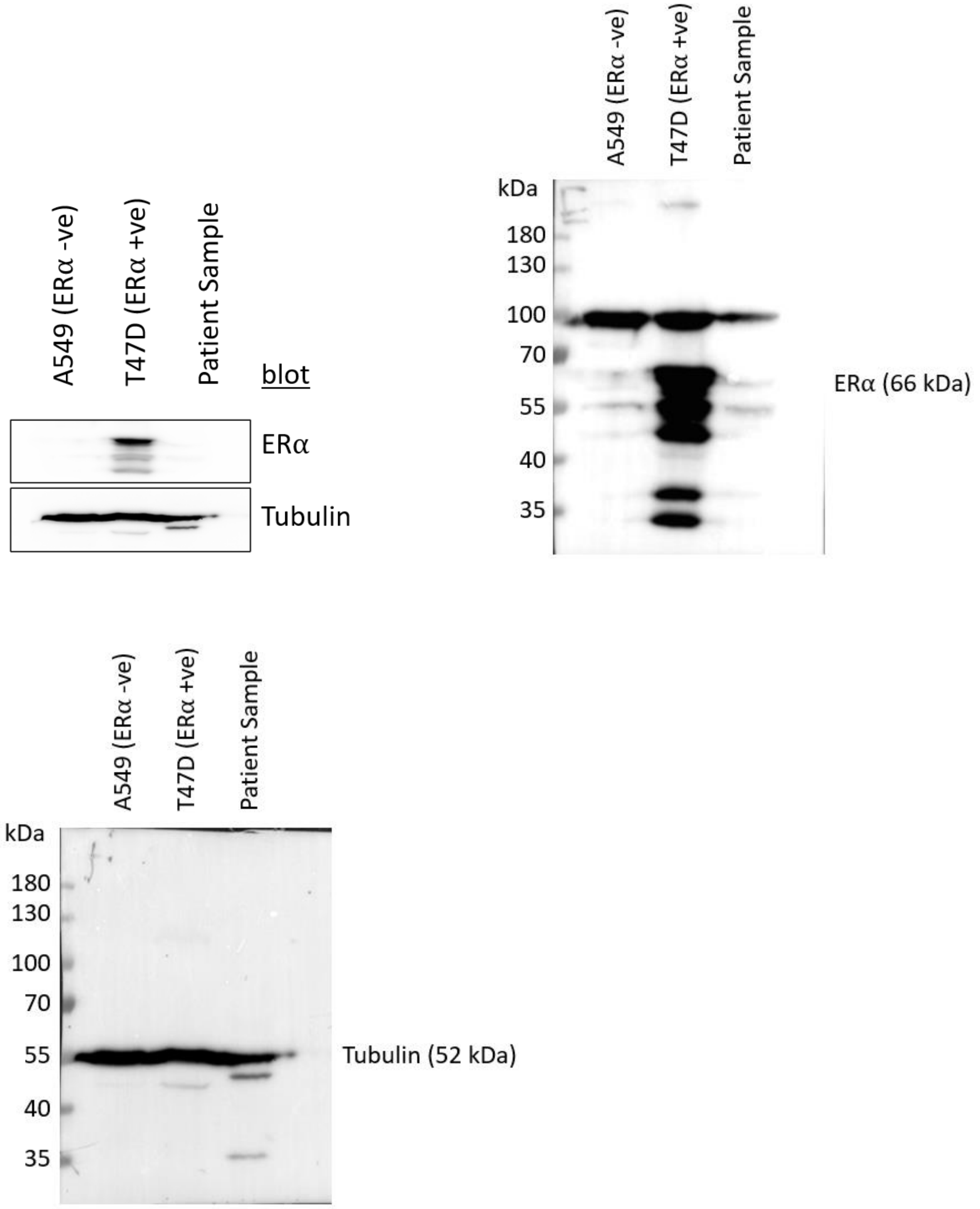

Supplementary Figure S1. Estrogen receptor α is undetectable in patient P3. Two breast cancer cell lines, A549 (ERα -ve) and T47D (ERα +ve), have been used as a negative and positive control correspondingly. Cells from A549, T47D and tumour tissue of patient P3 were lysed in RIPA buffer. Protein concentrations were measured and adjusted, and 60 µg of protein was loaded per lane. ERα and Tubulin expression were visualized using immunoblotting.

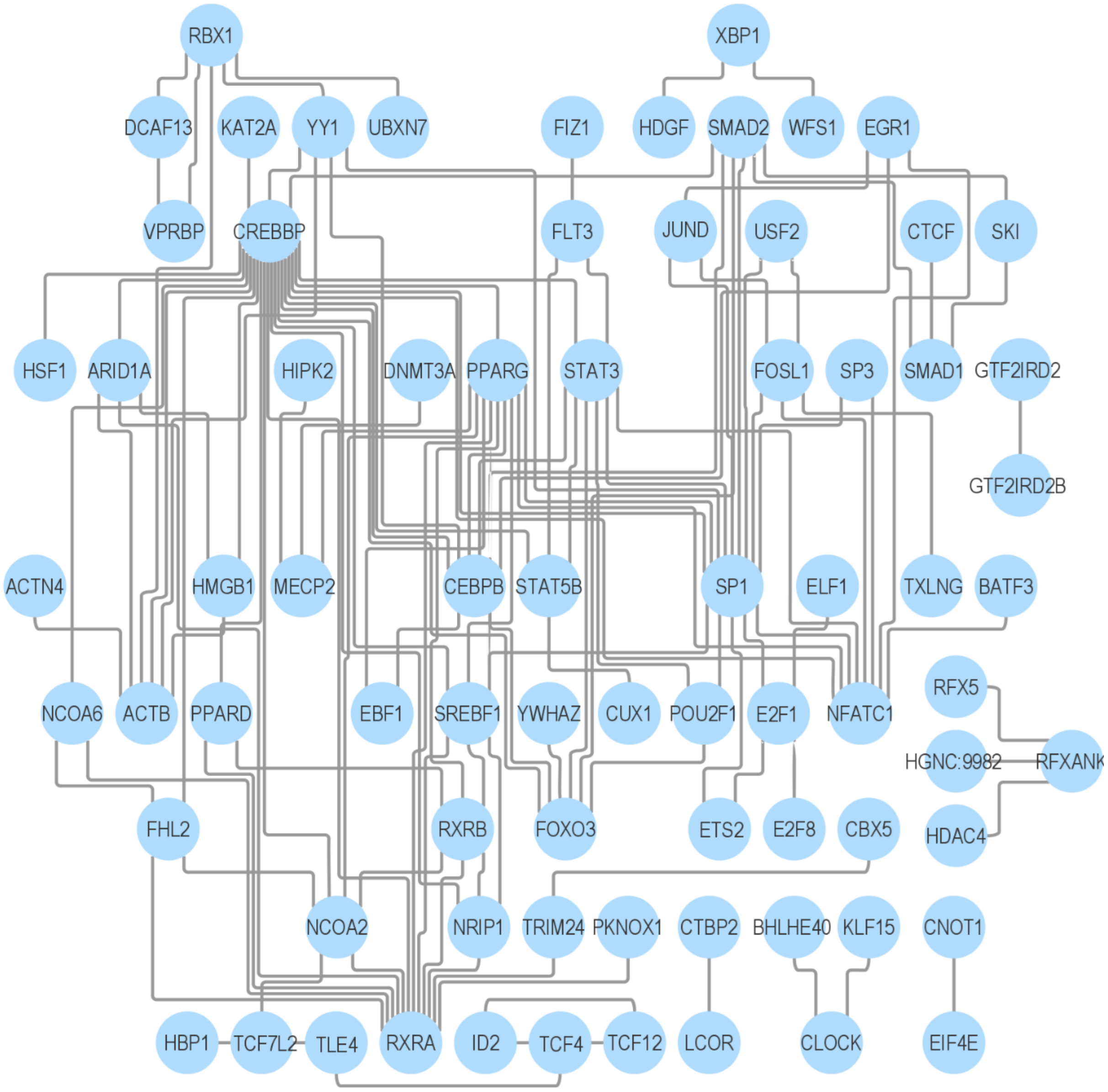

Supplementary Figure S2. Network of DNA-binding proteins encoded by genes marked by nucleosome gain at their promoters in tumour tissues. The network was constructed with Cytoscape 3.9.1 (Shannon et al. 2003) using STRING database (Szklarczyk et al. 2023), selecting entries with gene ontology term “DNA-binding”.

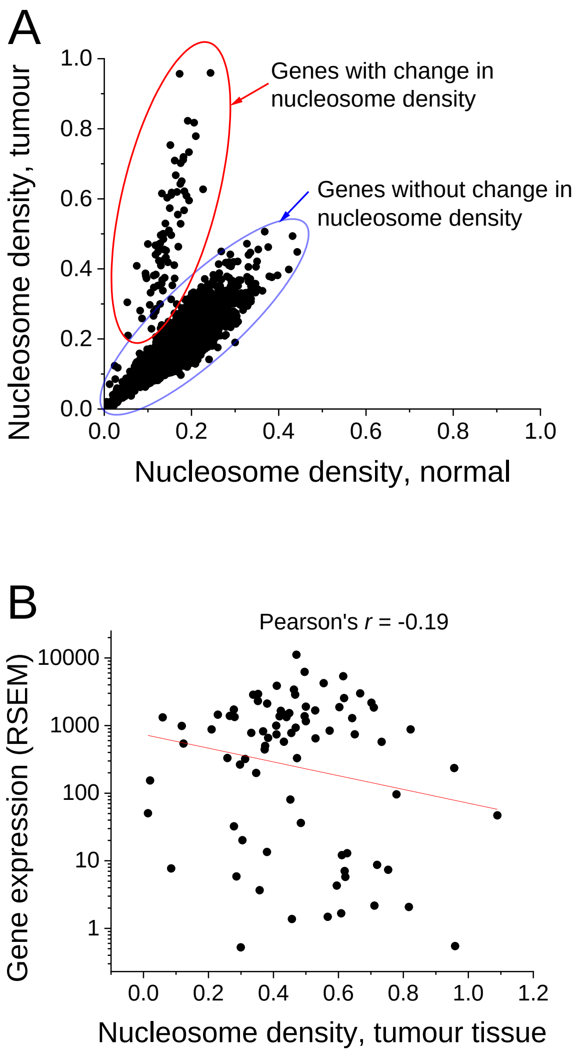

Supplementary Figure S3. Relationship between gene expression and nucleosome density in gene body. A) Scatter plot of nucleosome density in tumour and normal tissues shows a bimodal distribution, with a fraction of genes showing significant change of nucleosome density. B) Analysis of 88 genes which show >2-fold increase of nucleosome density across their gene body in tumour vs normal breast tissue of patient P2. Correlation between gene expression in breast cancer patients reported by the TCGA consortium and nucleosome density in tumour breast tissues of patient P2 from this study. Nucleosome density of a given gene was defined as the number of mapped MNase-seq DNA fragments divided by the gene length.

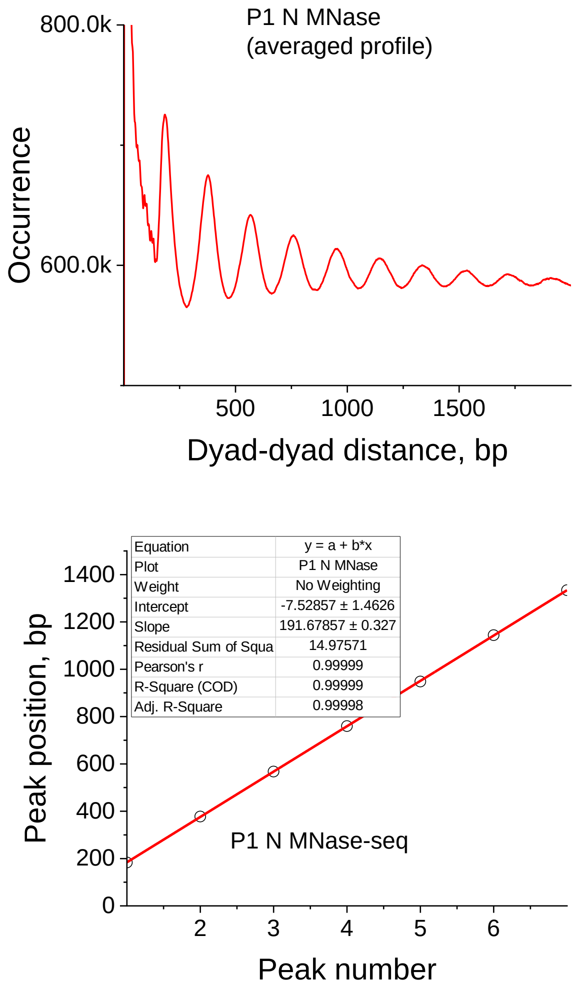

Supplementary Figure S4. Calculation of the genome-wide NRL for sample P1 N MNase. The averaged genome-wide distribution of occurrences of nucleosome dyad-dyad distances (top graph), and the linear regression calculating NRL based on the summits of the peaks of this distribution (bottom).

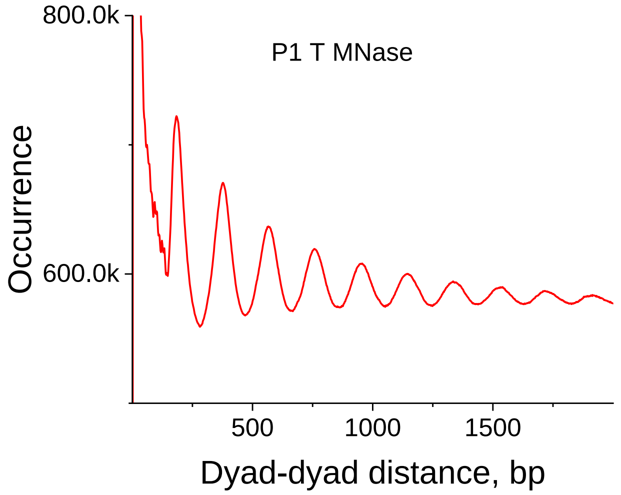

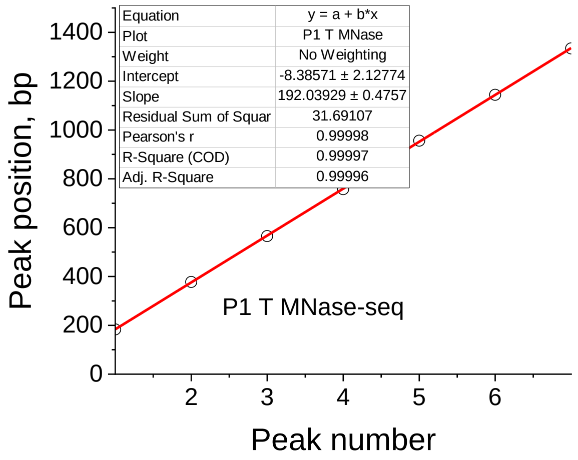

Supplementary Figure S5. Calculation of the genome-wide NRL for sample P1 T MNase. The averaged genome-wide distribution of occurrences of nucleosome dyad-dyad distances (top graph), and the linear regression calculating NRL based on the summits of the peaks of this distribution (bottom).

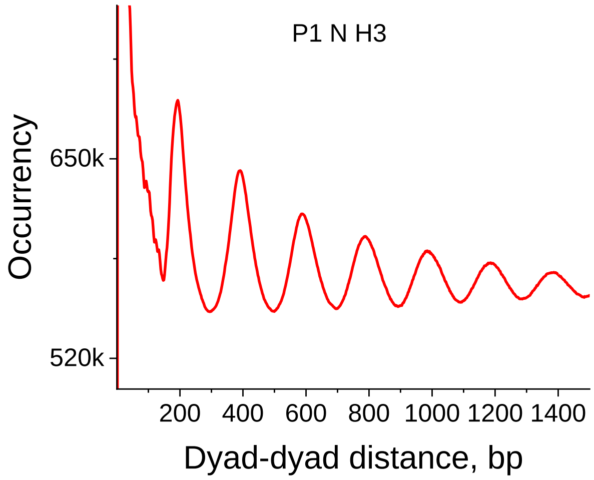

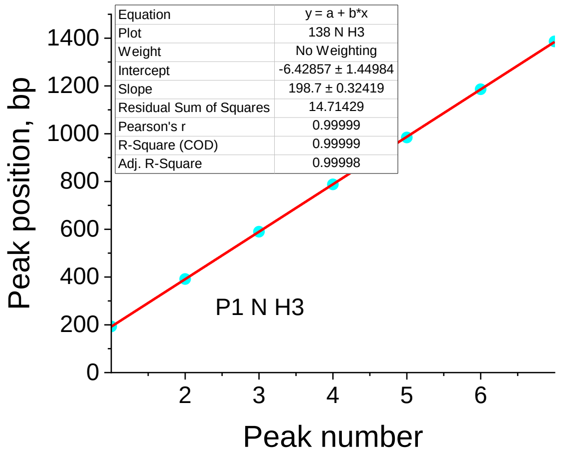

Supplementary Figure S6. Calculation of the genome-wide NRL for sample P1 N H3. The averaged genome-wide distribution of occurrences of nucleosome dyad-dyad distances (top graph), and the linear regression calculating NRL based on the summits of the peaks of this distribution (bottom).

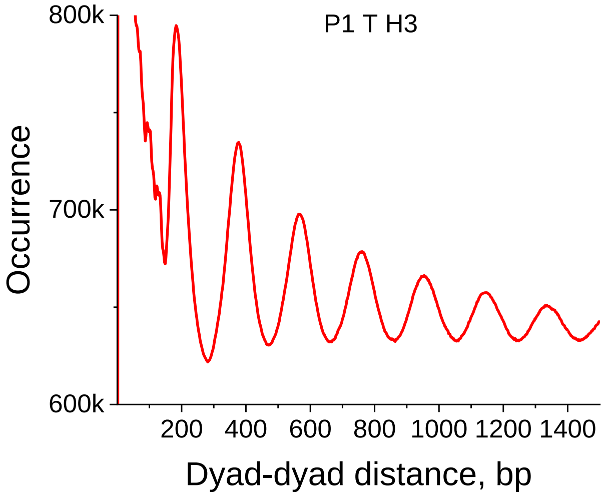

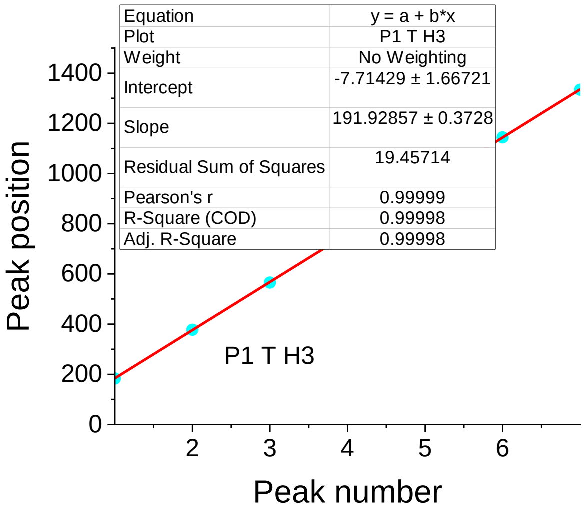

Supplementary Figure S7. Calculation of the genome-wide NRL for sample P1 T H3. The averaged genome-wide distribution of occurrences of nucleosome dyad-dyad distances (top graph), and the linear regression calculating NRL based on the summits of the peaks of this distribution (bottom).

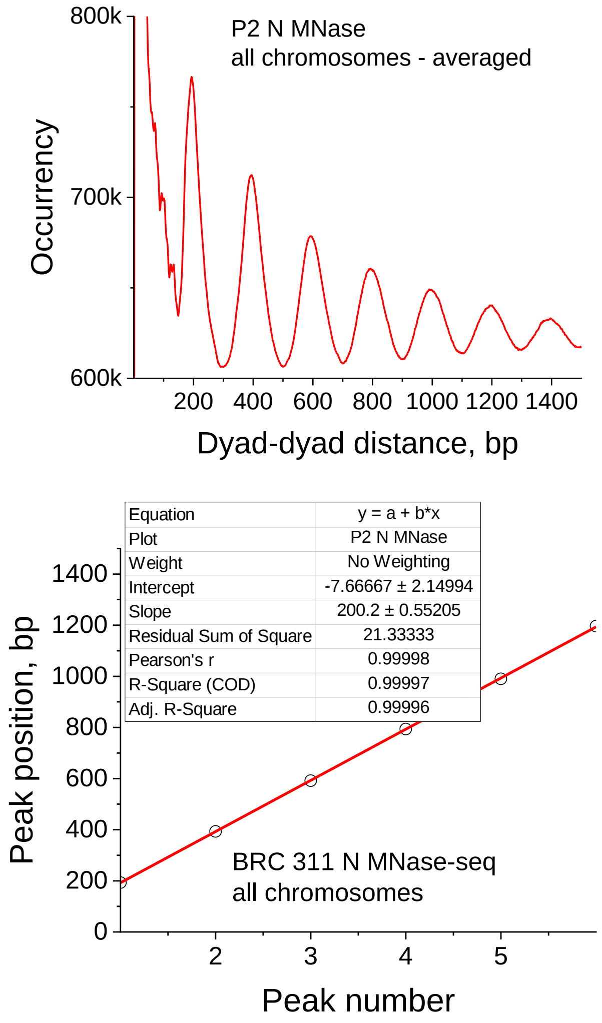

Supplementary Figure S8. Calculation of the genome-wide NRL for sample P2 N MNase. The averaged genome-wide distribution of occurrences of nucleosome dyad-dyad distances (top graph), and the linear regression calculating NRL based on the summits of the peaks of this distribution (bottom).

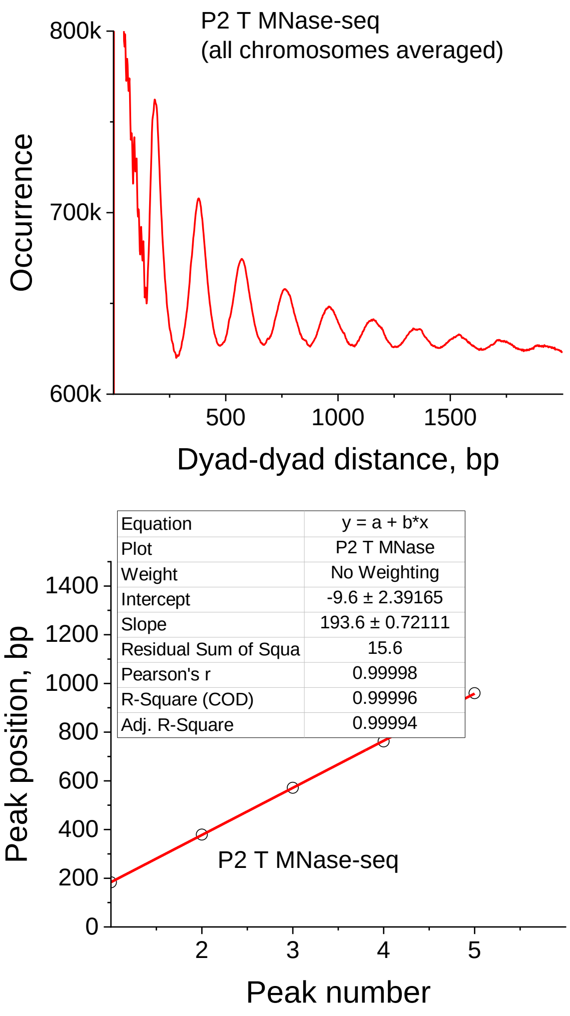

Supplementary Figure S9. Calculation of the genome-wide NRL for sample P2 T MNase. The averaged genome-wide distribution of occurrences of nucleosome dyad-dyad distances (top graph), and the linear regression calculating NRL based on the summits of the peaks of this distribution (bottom).

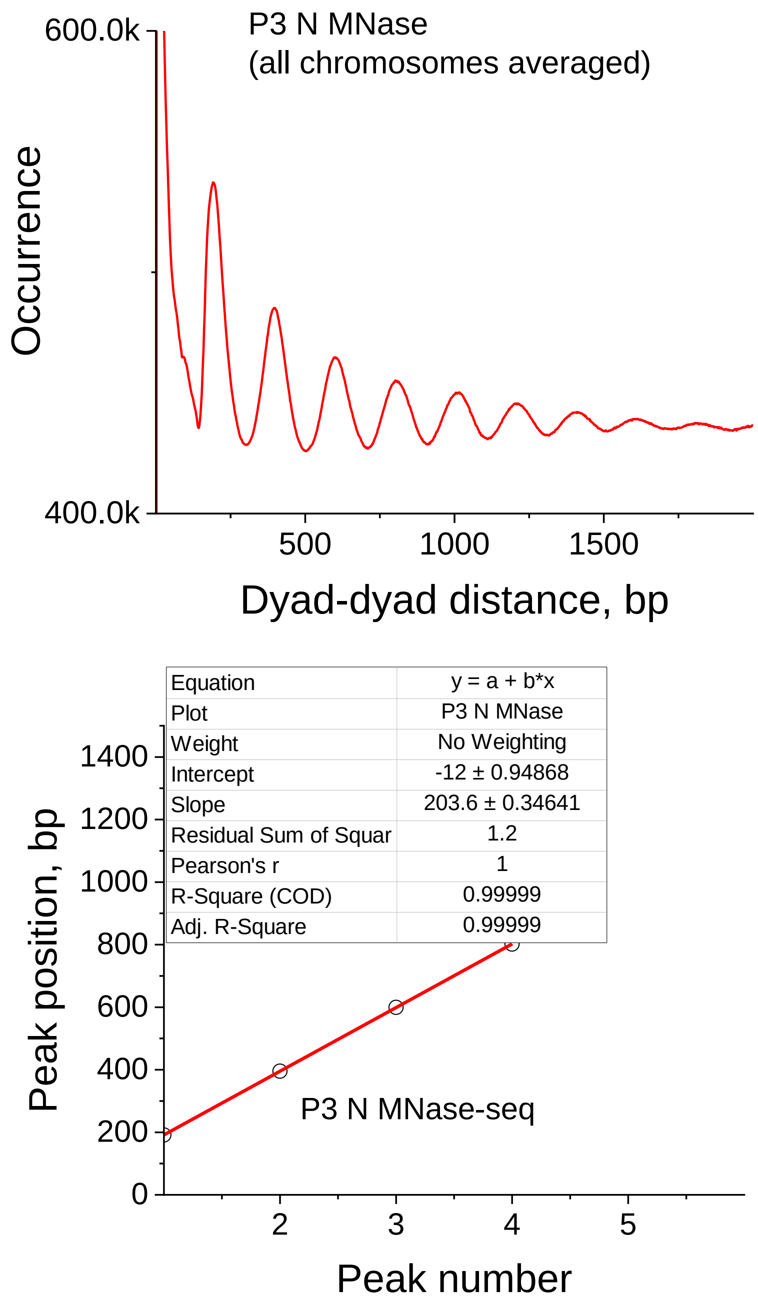

Supplementary Figure S10. Calculation of the genome-wide NRL for sample P3 N MNase. The averaged genome-wide distribution of occurrences of nucleosome dyad-dyad distances (top graph), and the linear regression calculating NRL based on the summits of the peaks of this distribution (bottom).

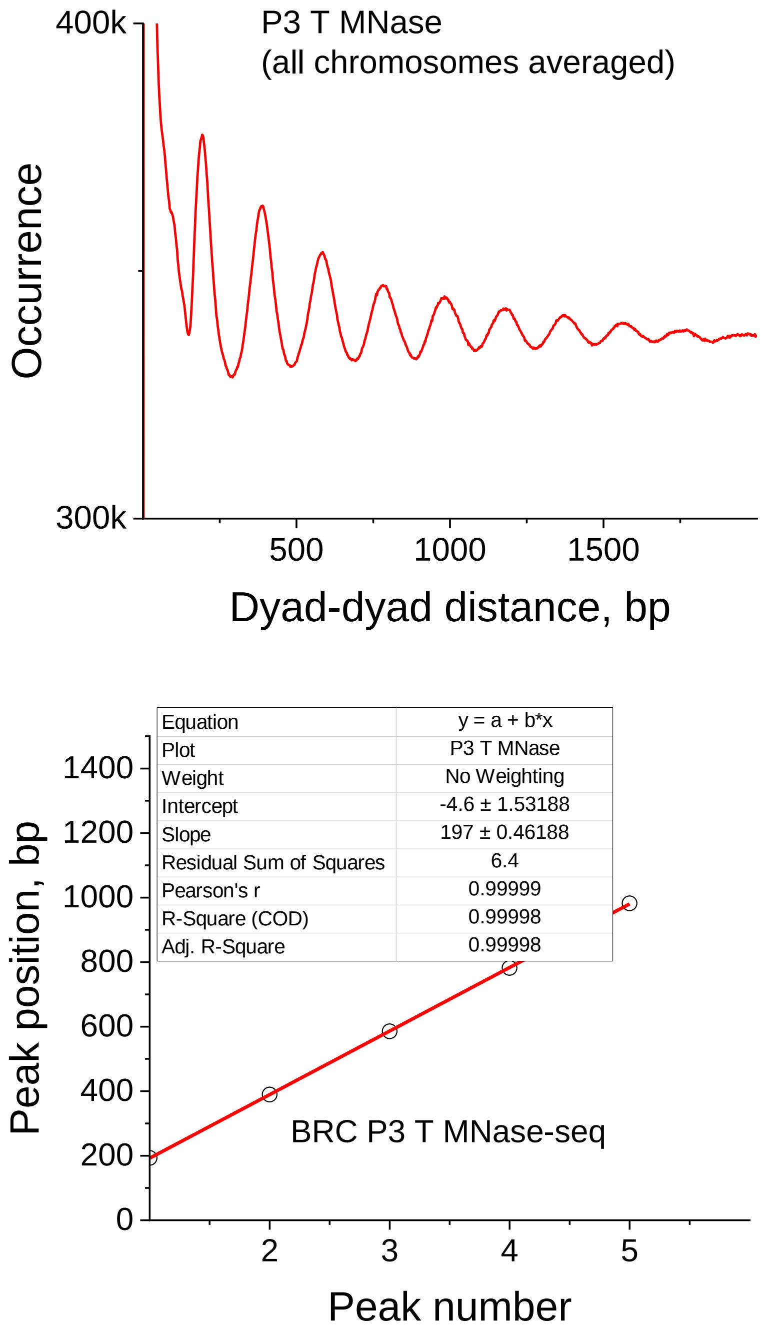

Supplementary Figure S11. Calculation of genome-wide NRL for sample P3 T MNase. The averaged genome-wide distribution of occurrences of nucleosome dyad-dyad distances (top graph), and the linear regression calculating NRL based on the summits of the peaks of this distribution (bottom).

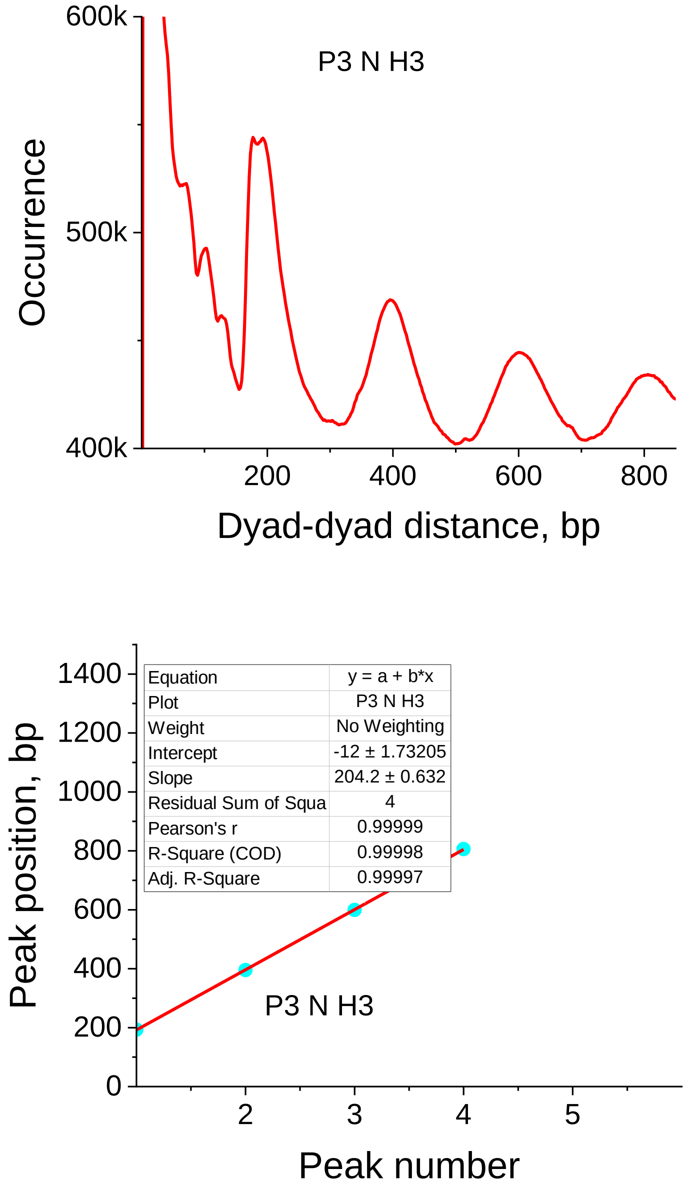

Supplementary Figure S12. Calculation of genome-wide NRL for sample P3 N H3. The averaged genome-wide distribution of occurrences of nucleosome dyad-dyad distances (top graph), and the linear regression calculating NRL based on the summits of the peaks of this distribution (bottom).

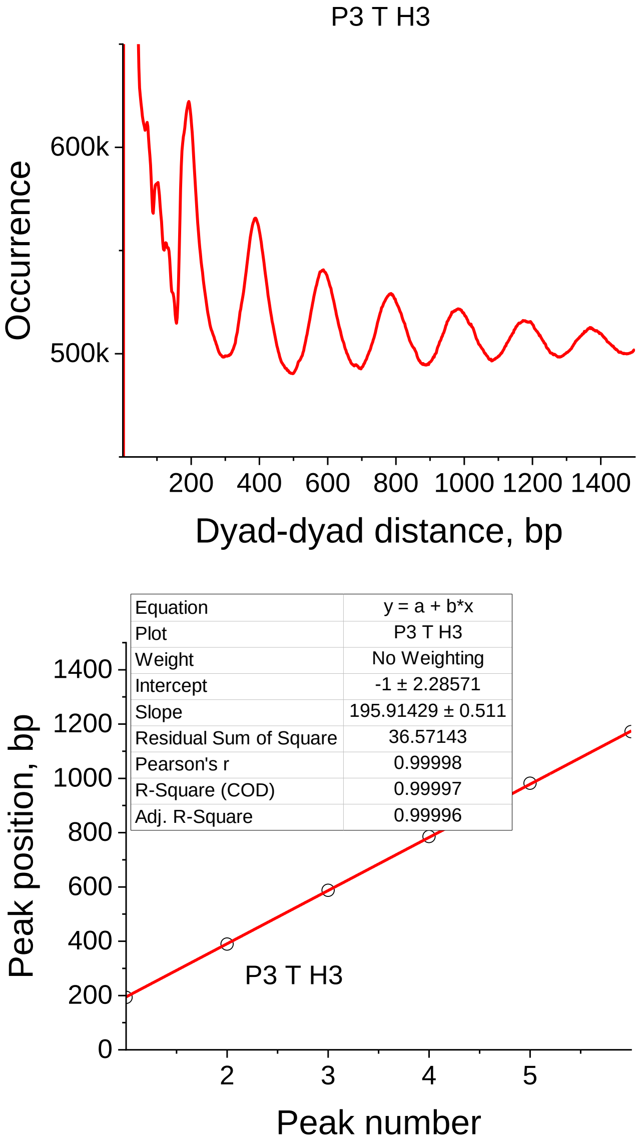

Supplementary Figure S13. Calculation of genome-wide NRL for sample P3 T H3. The averaged genome-wide distribution of occurrences of nucleosome dyad-dyad distances (top graph), and the linear regression calculating NRL based on the summits of the peaks of this distribution (bottom).

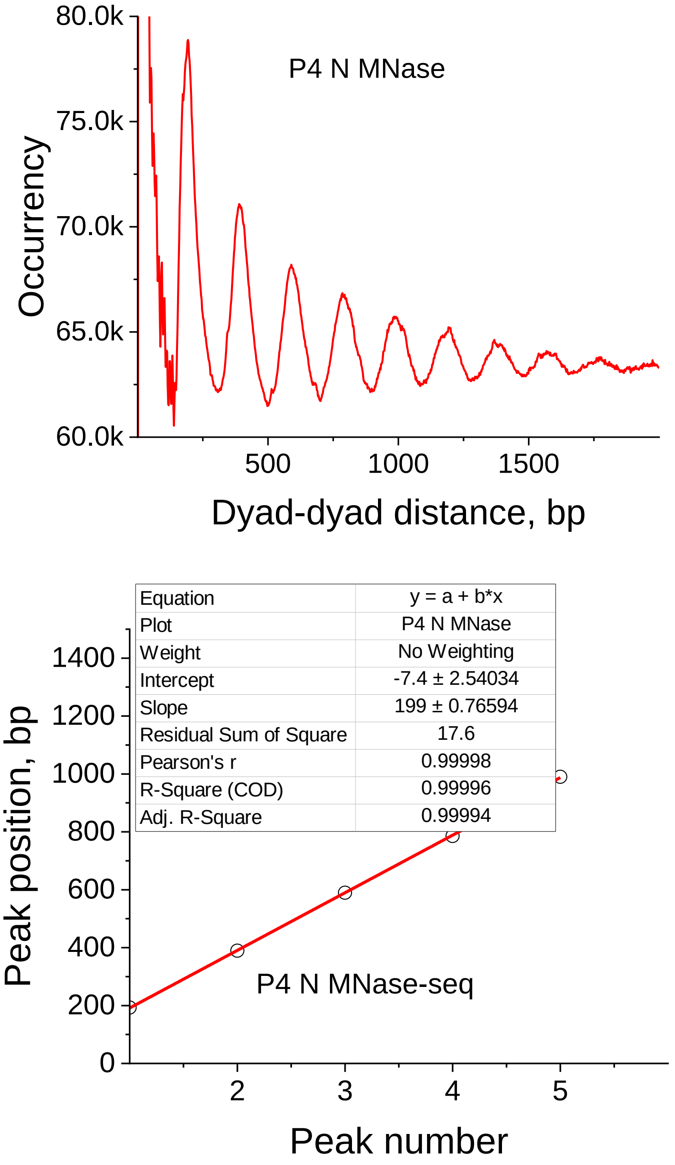

Supplementary Figure S14. Calculation of genome-wide NRL for sample P4 N MNase. The averaged genome-wide distribution of occurrences of nucleosome dyad-dyad distances (top graph), and the linear regression calculating NRL based on the summits of the peaks of this distribution (bottom).

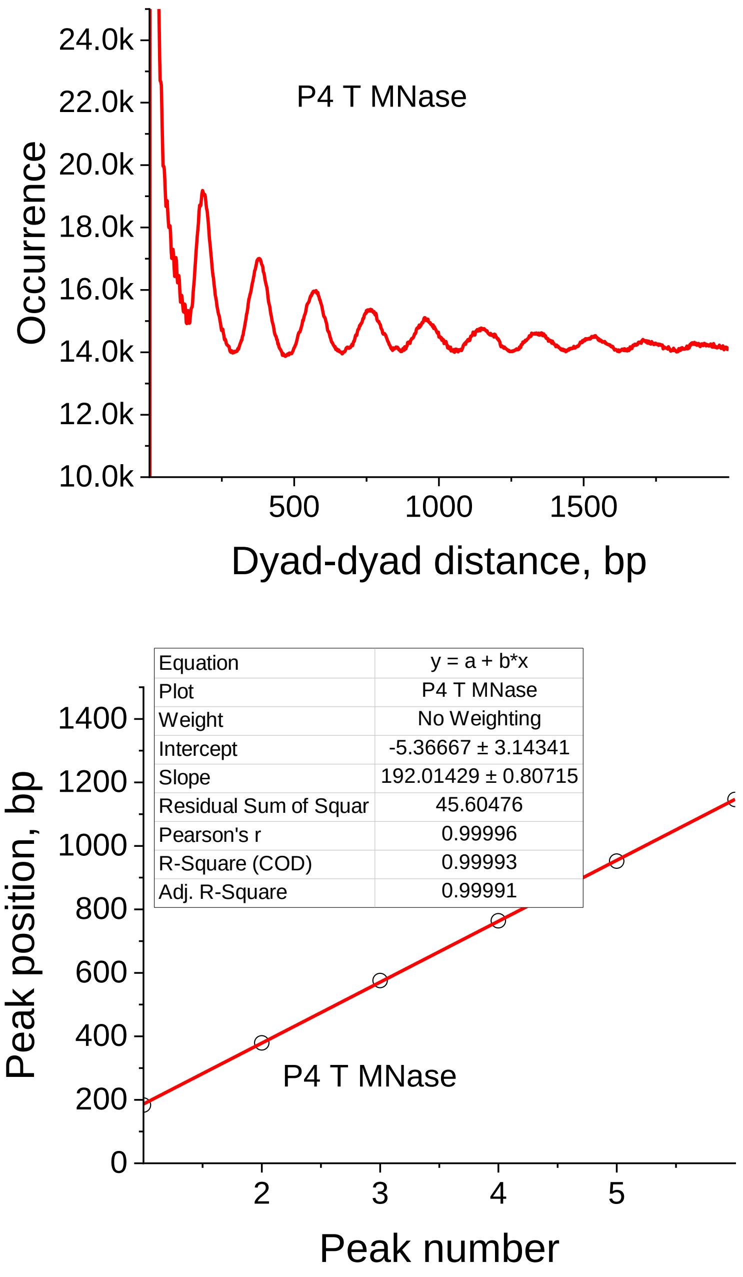

Supplementary Figure S15. Calculation of genome-wide NRL for sample P4 T MNase. The averaged genome-wide distribution of occurrences of nucleosome dyad-dyad distances (top graph), and the linear regression calculating NRL based on the summits of the peaks of this distribution (bottom).

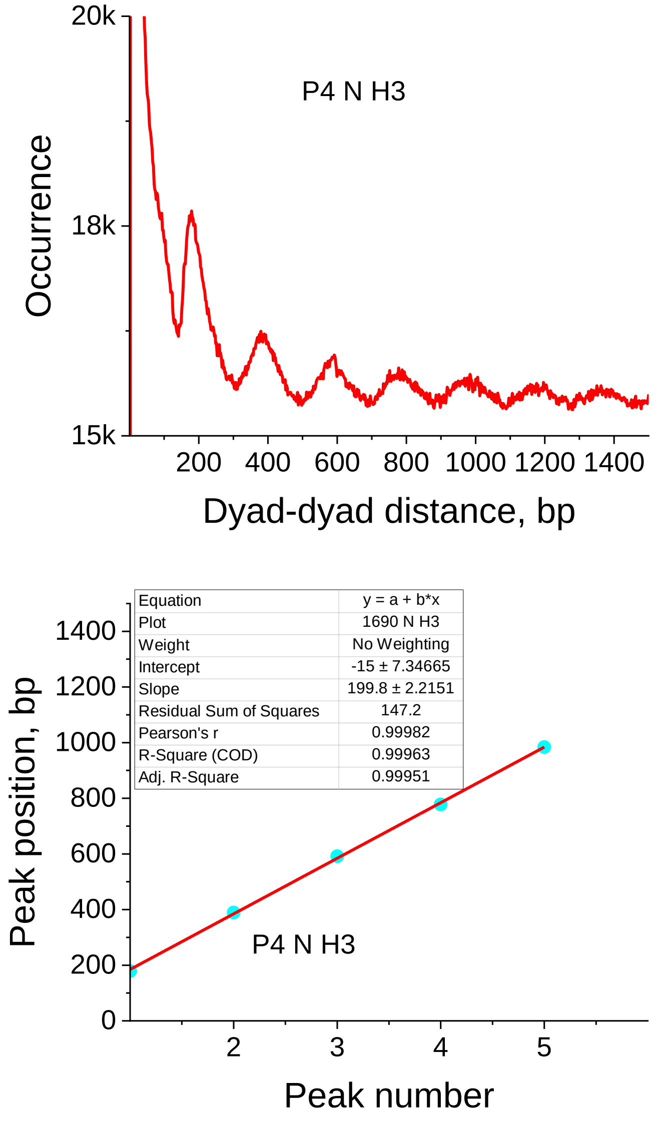

Supplementary Figure S16. Calculation of genome-wide NRL for sample P4 N H3. The averaged genome-wide distribution of occurrences of nucleosome dyad-dyad distances (top graph), and the linear regression calculating NRL based on the summits of the peaks of this distribution (bottom).

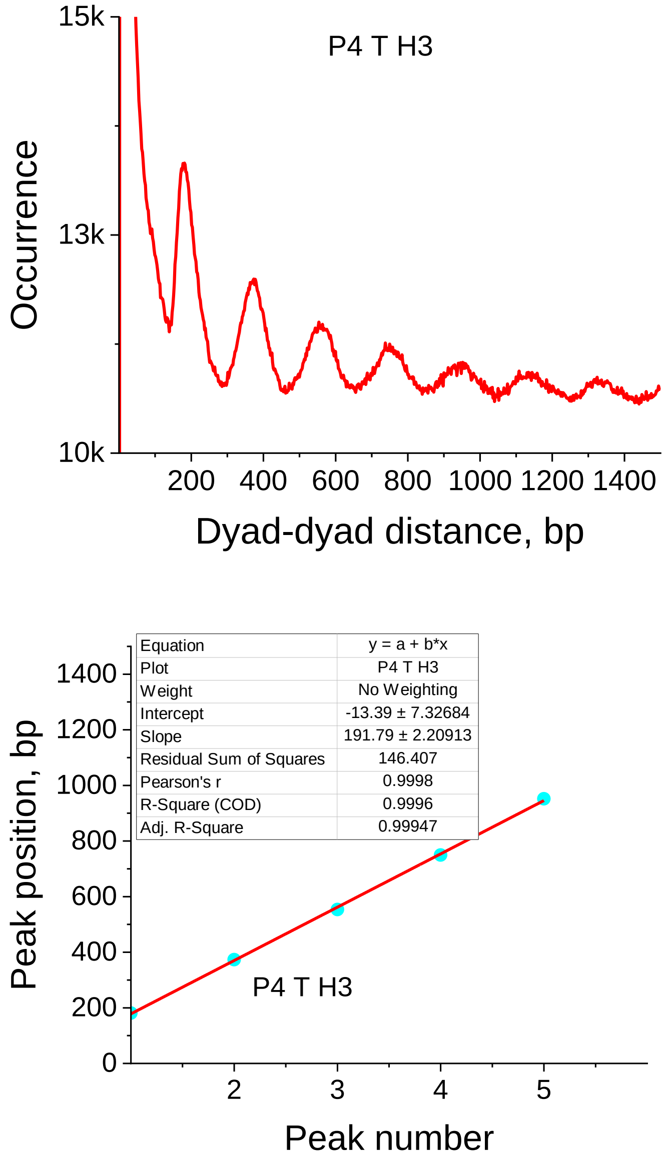

Supplementary Figure S17. Calculation of genome-wide NRL for sample P4 T H3. The averaged genome-wide distribution of occurrences of nucleosome dyad-dyad distances (top graph), and the linear regression calculating NRL based on the summits of the peaks of this distribution (bottom).

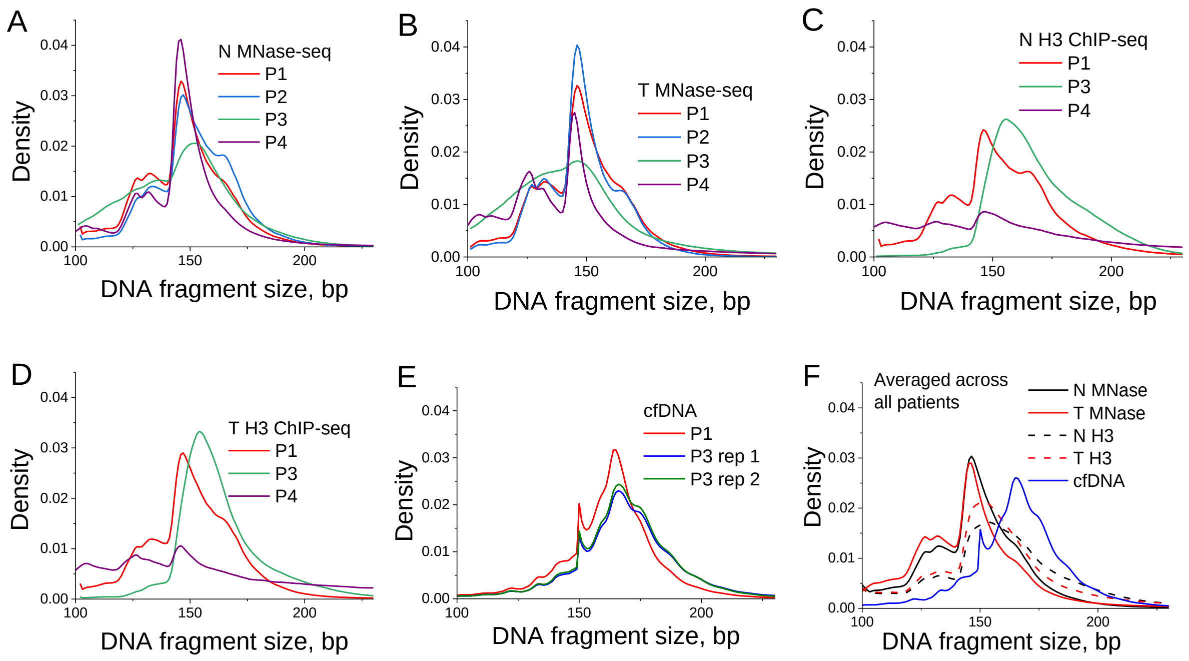

Supplementary Figure S18. Distribution of DNA fragments protected from digestion in individual patients and averaged across all patients. A) and B) MNase-seq; C) and D) MNase-assisted H3 ChIP-seq; E) cfDNA from blood plasma. N represent normal breast tissue samples, T represents tumour breast tissue. P1, P2, P3, P4 denote different patients. F) Same as A-E, averaged across all patients in each condition.

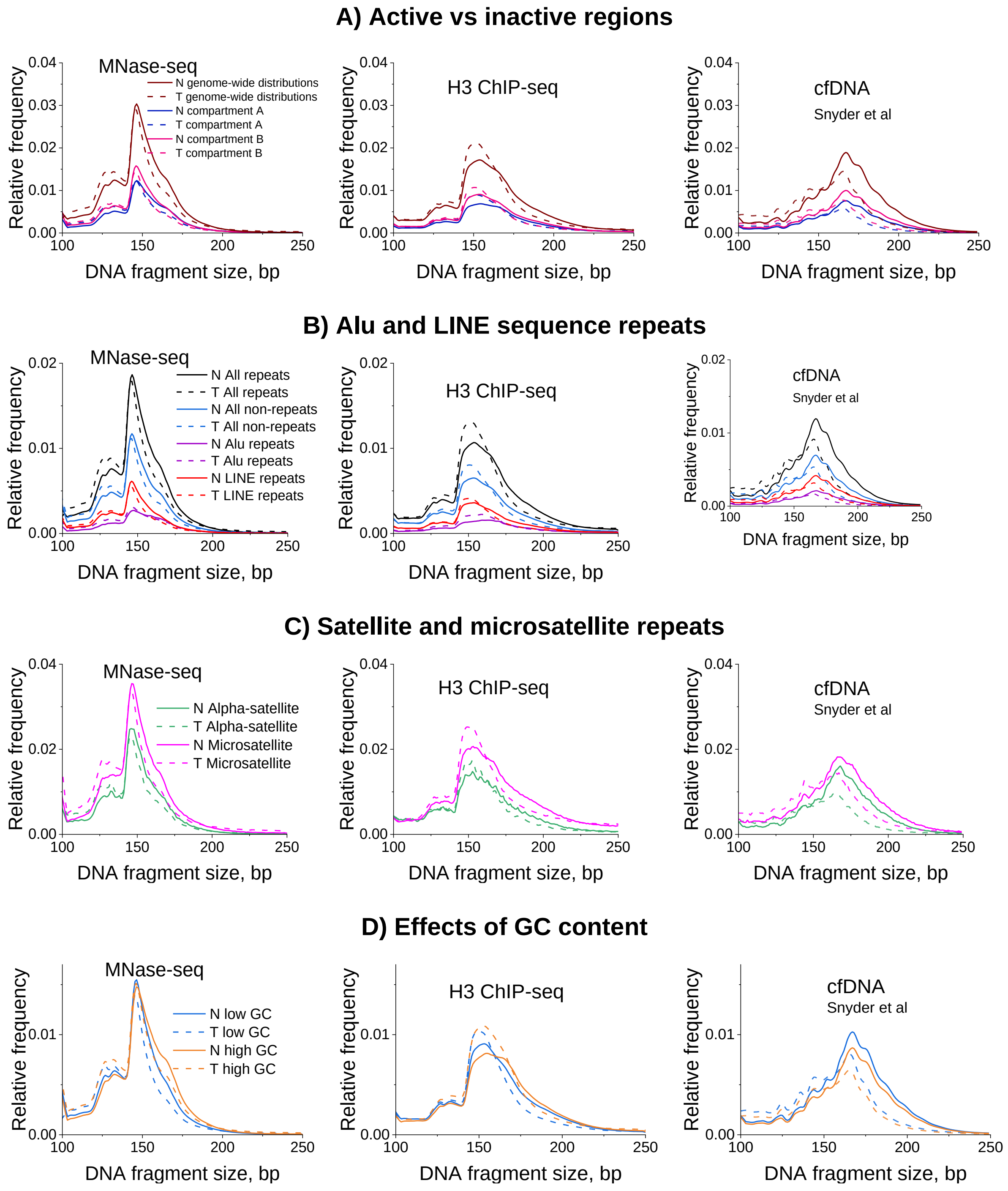

Supplementary Figure S19. Distribution of DNA fragments protected from digestion, averaged across all samples in a given condition based on MNase-seq, MNase-assisted H3 ChIP-seq from this study and cfDNA sequencing from Snyder et al (Snyder et al. 2016). A) DNA fragment size distributions inside A and B compartments in MCF7 cells and genome-wide. B-C) DNA fragment size distributions inside different types of repetitive and non-repetitive genomic regions indicated in the figure. D) DNA fragment size distributions inside 10-kb genomic regions with high and low GC content. The solid lines represent normal samples and dashed line represents tumour samples.

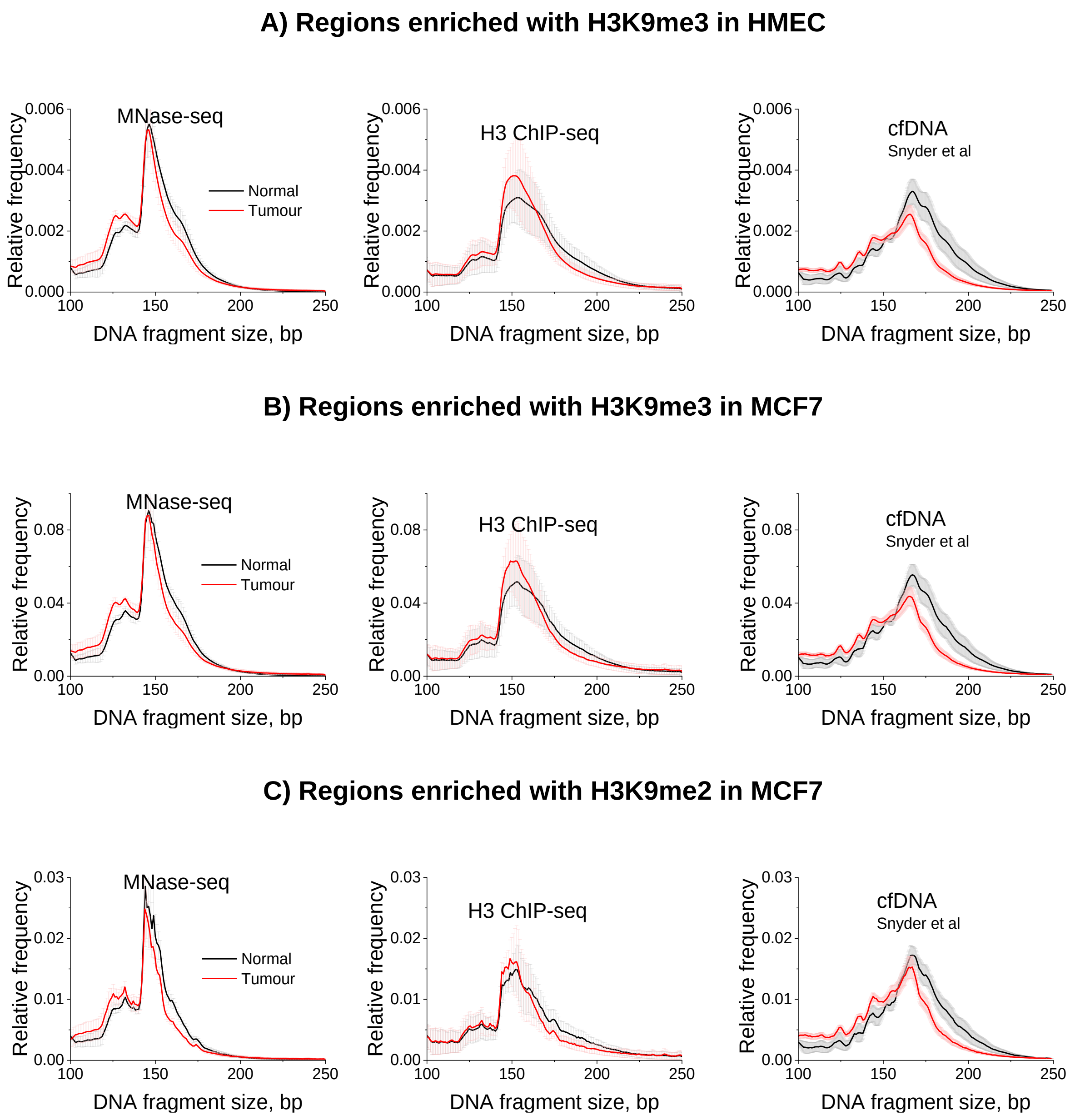

Supplementary Figure S20. Distribution of DNA fragment sizes protected from digestion inside genomic regions enriched with H3K9me2/3 histone modifications.

A) DNA fragment size distribution based on MNase-seq, MNase-assisted H3 ChIP-seq and cfDNA sequencing (Snyder et al. 2016) inside regions enriched with H3K9me3 in HMEC cells (healthy breast cells) (Zhang et al. 2020).

B) DNA fragment sizes based on MNase-seq, MNase-assisted H3 ChIP-seq and cfDNA sequencing inside regions enriched with H3K9me3 in MCF-7 cells (Dunham et al. 2012).

C) DNA fragment sizes based on MNase-seq, MNase-assisted H3 ChIP-seq and cfDNA sequencing inside regions enriched with H3K9me2 in MCF-7 cells (Dunham et al. 2012). The solid line corresponds to the averaged profile across all samples in a given condition. The red/grey clouds around the average line show the corresponding standard deviation of averaging.

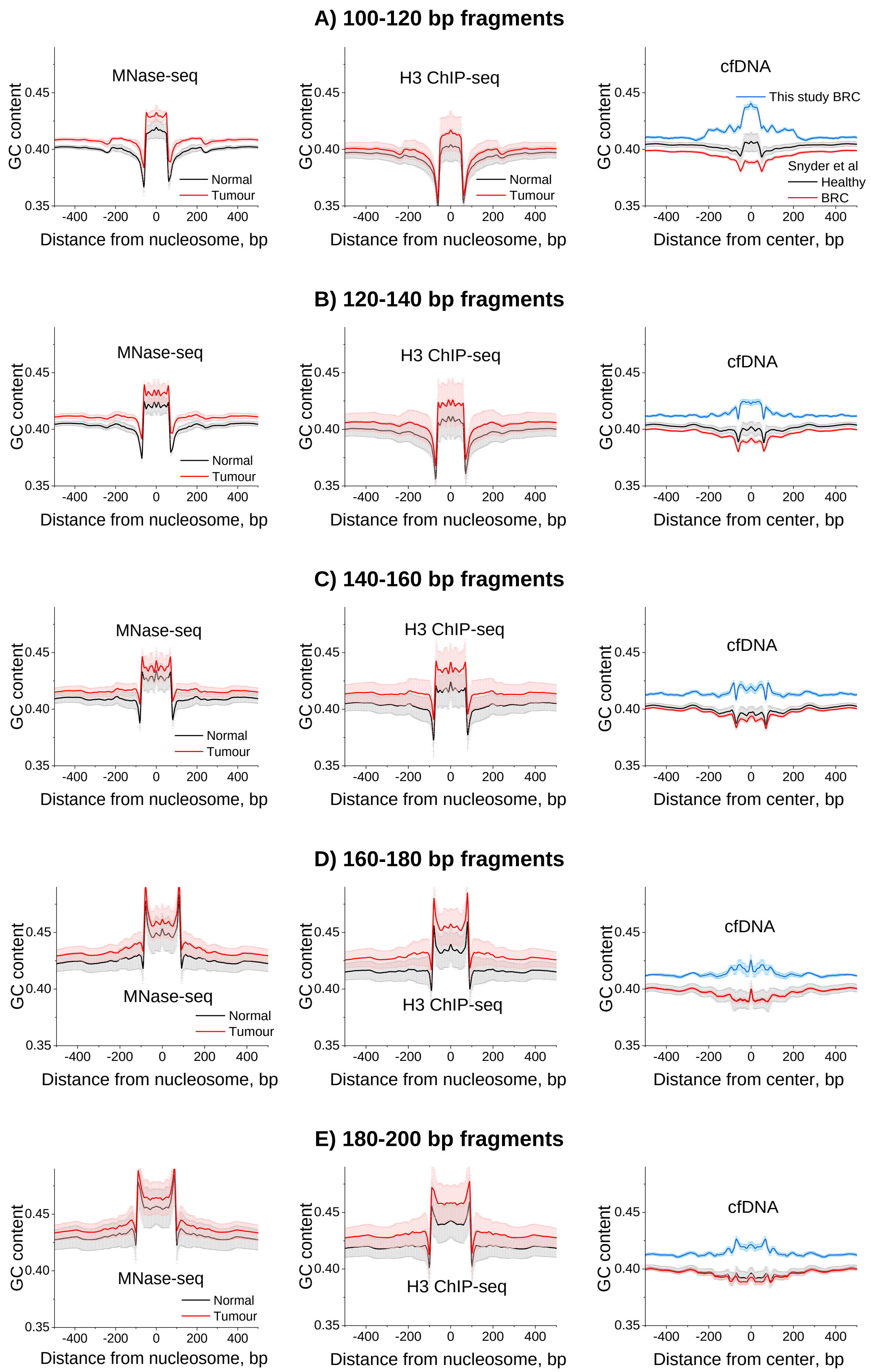
A) 100-120 bp fragments
B) 120-140 bp fragments
C) 140-160 bp fragments
D) 160-180 bp fragments
E) 180-200 bp fragments
MNase-seq
H3 ChIP-seq
cfDNA
GC content
0.35
0.40
0.45
-400
-200
0
200
400
Distance from nucleosome, bp
Distance from center, bp
Normal
Tumour
This study BRC
Snyder et al
Healthy
BRC

Supplementary Figure S21. GC profiles around DNA fragments protected from digestion in normal/breast cancer tissues and cfDNA genome-wide. Left column: MNase-seq experiments. Middle column: MNase-assisted H3 ChIP-seq. Right column: cfDNA reported by Snyder et al., 2016 (black, healthy controls and red, BRC patients) and cfDNA from this study (BRC patients, blue). DNA fragment sizes were selected in the following groups: 100-120 bp (A), 120-140 bp (B), 140-160 bp (C), 160-180 bp (D), 180-200 bp (E). The solid line corresponds to the averaged profile across all samples in a given condition. The red/grey/blue clouds around the average line show the corresponding standard deviation of averaging.

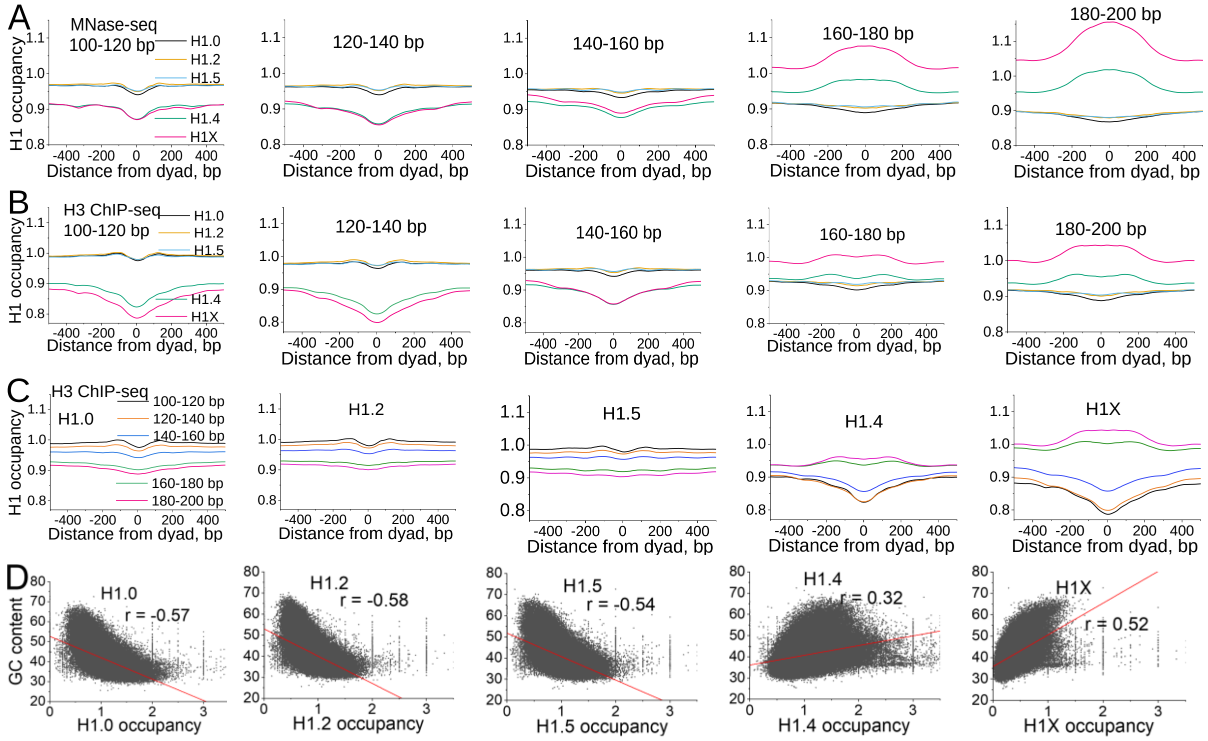

Supplementary Figure S22. **Occupancy of linker histone H1 variants around DNA fragments protected from MNase digestion in breast cancer tissues inside genes.**

A) MNase-seq. B) MNase-assisted H3 ChIP-seq. The profiles are shown separately for DNA fragment sizes in the following groups: 100-120 bp, 120-140 bp, 140-160 bp, 160-180 bp,180-200 bp, for the following H1 variants were selected: H1.0 (black), H1.2 (orange), H1.5 (blue), H1.4 (green) and H1X (pink).

C) Aggregate profiles of occupancy of H1 variants around nucleosome dyads calculated for different DNA fragment sizes in MNase-assisted H3 ChIP-seq. The profiles are shown separately for DNA fragment sizes in the following groups: 100-120 bp (black), 120-140 bp (orange),140-160 bp (blue),160-180bp (green) and 180-200 bp (pink).

D) Correlation of occupancy of H1 variants with GC content as in Figure 3A, but calculated inside gene bodies, with each point corresponding to one gene.

Supplementary Table S1. Clinical information of patients P1 and P2.

Patient ID	Age	Meno stats	Tumour size	Lymph nodes	ER	PR	Stage	Grade	Diagnosis
P1	84	Post	25	Neg	7	3	II	1	Invasive ductal carcinoma
P2	60	Post	9	Pos	8	7	II	2	Mixed Invasive Ductal and Lobular Carcinoma

Supplementary Table S2. Numbers of DNA fragments used in the analysis. Sample names are composed of the patient codes, P1, P2, P3, P4; N – normal breast tissue; T – tumour breast tissue; cfDNA – cell-free DNA from the corresponding patient.

Sample name	Total reads	Uniquely mapped	Reads 120-180 bp
P1 N MNase-seq	738685119	581546291	434837001
P1 T MNase-seq	690415722	546197304	406009852
P1 N MNase-H3-seq	799317821	593479462	405213397
P1 T MNase-H3-seq	726535624	574236019	417837382
P1 cfDNA	605965018	305961716	455380323
P2 N MNase-seq	740166473	589186960	442219740
P2 T MNase-seq	721399958	582290379	450655870
P3 N MNase-seq	719309252	552228048	367379663
P3 T MNase-seq	734554908	500718271	311730314
P3 N MNase-H3-seq	704821706	531125999	313518972
P3 T MNase-H3-seq	825637262	578205688	386531315
P3 cfDNA 1	802041150	420816769	446372537
P3 cfDNA 2	854862937	453457506	515977495
P4 N MNase-seq	198922080	147359246	114522142
P4 T MNase-seq	176299238	107963750	65775814
P4 N MNase-H3-seq	191369339	136183830	51150348
P4 T MNase-H3-seq	171247792	122174984	50702774

Supplementary Table S3. Resequenced reads from the same samples as in Table S2.

Sample name	Uniquely mapped	Reads 120-180 bp
P1 N MNase-seq	921526763	720919673
P1 T MNase-seq	785058607	614106072
P1 N H3 ChIP-seq	770087909	565896992
P1 cfDNA	590122263	455380323
P2 N MNase-seq	851602991	691252262
P2 T MNase-seq	906470171	735504524
P3 N MNase-seq	729198144	506009505
P3 T MNase-seq	1166043773	710577513
P3 cfDNA 1	740668098	446372537
P3 cfDNA 2	808026801	515977495

Supplementary Table S4. Numbers of "stable", "common", "shifted", "lost" and "gained" DNA fragments protected from MNase digestion with sizes 120-180 bp and 160-180 bp.

	120-180 bp	160-180 bp
**Stable (tumour tissue)**	418,131,153	72,266,802
**Stable (normal tissue)**	403,536,177	107,470,477
**Common**	308,997,468	36,994,858
**Shifted**	9,460,589	6,529,484
**Lost**	57,210	2,310,979
**Gained**	49,964	337,940

Supplementary Table S5. List of genes encoding DNA-binding proteins marked by gained nucleosomes at their promoters.

ACTB	EIF4E	KLF15	RFX1	TFEC	ZNF568
ACTN4	ELF1	KLF16	RFX2	TGIF2	ZNF574
AKAP8	ELK4	KLF7	RFX5	THAP11	ZNF596
ARID1A	ETS2	LCOR	RFXANK	TLE4	ZNF615
ATMIN	ETV4	LMO2	RREB1	TPRX1	ZNF618
BACH1	FHL2	LMX1B	RXRA	TRIM24	ZNF627
BACH2	FIZ1	MAD2L2	RXRB	TXLNG	ZNF658
BARHL2	FLT3	MAFA	SALL1	UBXN7	ZNF689
BATF3	FOSL1	MDFIC	SALL3	UNCX	ZNF691
BCL10	FOXA2	MECP2	SETD3	USF2	ZNF703
BCL11B	FOXB2	MEIS3	SIM2	WFS1	ZNF775
BCL6B	FOXC1	MIXL1	SIX5	XBP1	ZNF829
BHLHA15	FOXD4L5	MXD1	SKI	YWHAZ	ZNF837
BHLHE40	FOXE1	MXD4	SLC2A4RG	YY1	ZNF84
CARF	FOXF2	MXI1	SMAD1	ZBTB1	ZNF845
CBX5	FOXO3	MYBL2	SMAD2	ZBTB14	ZNF880
CEBPB	GATA5	MYCL	SNAI3	ZBTB18	ZNHIT3
CGGBP1	GBX1	NCOA2	SOX1	ZBTB2	ZSCAN10
CIC	GSC2	NCOA6	SOX10	ZBTB20	ZSCAN26
CLOCK	GTF2I	NEUROG2	SOX18	ZBTB25	
CNOT1	GTF2IRD2	NFATC1	SOX8	ZBTB4	
CREBBP	GTF2IRD2B	NFIB	SP1	ZBTB48	
CREBRF	HBP1	NFIC	SP3	ZBTB7A	
CTBP2	HDAC4	NPAS3	SP4	ZFHX3	
CTCF	HDGF	NR1D2	SPEN	ZFP62	
CTCFL	HES3	NRF1	SREBF1	ZFPM2	
CTDP1	HIF1AN	NRIP1	STAT3	ZNF107	
CUX1	HIPK2	NUCKS1	STAT5B	ZNF182	
CXXC5	HIVEP1	PAX2	SUB1	ZNF316	
DACT1	HMGB1	PITX1	TBX2	ZNF322	
DCAF1	HOXC10	PKNOX1	TCERG1	ZNF337	
DCAF13	HOXC8	PLAG1	TCF12	ZNF362	
DGKQ	HSF1	POU2F1	TCF4	ZNF391	
DHX33	ID2	POU2F3	TCF7L2	ZNF395	
DNMT3A	INSM1	POU3F3	TCFL5	ZNF423	
DOT1L	IRX3	PPARD	TEAD3	ZNF48	
E2F1	JUND	PPARG	TEAD4	ZNF496	
E2F8	KAT2A	PRDM15	TFAP2E	ZNF516	
EBF1	KCNIP3	PRDM4	TFAP4	ZNF521	
EGR1	KLF13	RBX1	TFEB	ZNF524	

Supplementary Table S6. List of genes marked by increased nucleosome density across the gene body in tumour tissue.

ADAM18	CHRNB3	GUSB	PLAT	SMIM19	ZC3H11B
ADAM2	CRCP	HGSNAT	PLEKHA2	SPDYE16	ZNF107
ADAM32	DDHD2	HSPB1	PLPBP	SPDYE5	ZNF117
ADAM9	DKK4	HTRA4	PLPP5	SRRM3	ZNF138
ADGRA2	DTX2	IDO1	POLB	SSC4D	ZNF273
ADRB3	EIF4EBP1	IDO2	POM121C	STYXL1	ZNF679
AP3M2	ERLIN2	IKBKB	POMK	TACC1	ZNF680
ASH2L	ERV3-1	KAT6A	POR	TCIM	ZNF703
ASL	ETDA	KCNU1	POTEA	THAP1	ZNF727
BAG4	FNTA	LETM2	RAB11FIP1	TM2D2	ZNF735
C8orf86	GINS4	LSM1	RCC1L	TMEM120A	ZNF736
CCL24	GOLGA7	MDH2	RHBDD2	TMEM191B	ZNF92
CCL26	GOT1L1	MMP23B	RNF170	UPK3B	ZP3
CEBPB	GPAT4	NKX6-3	SFRP1	VKORC1L1	
CHRNA6	GTF2IRD2B	NSD3	SLC20A2	YWHAG	

Supplementary Table S7. Genome-wide NRL values in normal and tumour breast tissues in the samples from this study.

Sample ID	NRL in normal tissue, bp	Standard error, bp (normal)	NRL in tumour tissue, bp	Standard error, bp (tumour)
P1 MNase	191.7	0.3	192.0	0.5
P2 MNase	200.2	0.6	193.6	0.7
P3 MNase	203.6	0.3	197.0	0.5
P4 MNase	199.0	0.8	192.0	0.8
P1 H3	198.7	0.3	191.9	0.4
P3 H3	204.2	0.6	195.9	0.5
P4 H3	199.8	2.2	191.8	2.2

Supplementary Table S8. Genome-wide NRL values in cfDNA samples from this study

Sample ID	NRL, bp	Standard error, bp
P1 cfDNA	193.9	0.5
P3 cfDNA replicate 1	192.1	0.8
P3 cfDNA replicate 2	192.3	0.3

Supplementary Table S9. Genome-wide NRL values in human embryonic stem cells (hESCs) calculated based on the MNase-seq dataset GSE49140 by Yazdi et al (Yazdi et al. 2015). The corresponding accession numbers in the Short Read Archive (SRA) are indicated in the table.

SRA sample ID	NRL, bp	Standard error, bp
SRR942485	194.8	1.6
SRR942489	195.2	1.0
SRR942490	195.6	0.6
SRR942492	195.1	0.8
SRR942493	194.6	0.6
SRR942497	194.8	0.9
SRR942498	193.4	1.3

Supplementary Table S10. Genome-wide NRL values in MCF-7 cells calculated based on MNase-seq datasets GSE77526* and GSE51097 by Shimbo et al (Shimbo et al. 2013).

**Sample ID**	**NRL, bp**	**Standard error, bp**
GSE51097, replicate 1	192.1	0.2
GSE51097, replicate 2	194.9	0.2
GSE77526, 0.1 U MNase	192.6	1.3
GSE77526, 0.2 U MNase	192.0	1.2
GSE77526, 0.4 U MNase	194.9	1.9
GSE77526, 0.8 U MNase	194.6	0.6
GSE77526, 1.6 U MNase	194.8	0.4
GSE77526, 4 U MNase	195.4	0.7
GSE77526, 8 U MNase	194.8	0.4
GSE77526, 16 U MNase	195.8	1.0

*This dataset contains samples digested with different amounts of MNase units indicated in the table

Supplementary Table S11. Genome-wide NRL values in T47D cells calculated based on the MNase-seq dataset GSE74308 by Lavender et al (Lavender et al. 2016). The corresponding accession numbers in the Short Read Archive (SRA) are indicated in the table.

**SRA sample ID**	**NRL, bp**	**Standard error, bp**
SRR2774668	187.65	0.2
SRR2774669	189.03	1.2
SRR2774670	187.09	1.2
SRR2774671	187.92	1.2
SRR2774672	189.03	1.2

Supplementary References

Dunham I Kundaje A Aldred SF Collins PJ Davis CA Doyle F Epstein CB Frietze S Harrow J Kaul R et al. 2012. An integrated encyclopedia of DNA elements in the human genome. *Nature* **489**: 57-74.

Lavender CA, Cannady KR, Hoffman JA, Trotter KW, Gilchrist DA, Bennett BD, Burkholder AB, Burd CJ, Fargo DC, Archer TK. 2016. Downstream Antisense Transcription Predicts Genomic Features That Define the Specific Chromatin Environment at Mammalian Promoters. *PLoS genetics* **12**: e1006224.

Shannon P, Markiel A, Ozier O, Baliga NS, Wang JT, Ramage D, Amin N, Schwikowski B, Ideker T. 2003. Cytoscape: a software environment for integrated models of biomolecular interaction networks. *Genome Res* **13**: 2498-2504.

Shimbo T, Du Y, Grimm SA, Dhasarathy A, Mav D, Shah RR, Shi H, Wade PA. 2013. MBD3 localizes at promoters, gene bodies and enhancers of active genes. *PLoS genetics* **9**: e1004028.

Snyder MW, Kircher M, Hill AJ, Daza RM, Shendure J. 2016. Cell-free DNA comprises an in vivo nucleosome footprint that informs its tissues-of-origin. *Cell* **164**: 57-68.

Szklarczyk D, Kirsch R, Koutrouli M, Nastou K, Mehryary F, Hachilif R, Gable AL, Fang T, Doncheva NT, Pyysalo S et al. 2023. The STRING database in 2023: protein-protein association networks and functional enrichment analyses for any sequenced genome of interest. *Nucleic Acids Res* **51**: D638-D646.

Yazdi PG, Pedersen BA, Taylor JF, Khattab OS, Chen YH, Chen Y, Jacobsen SE, Wang PH. 2015. Nucleosome Organization in Human Embryonic Stem Cells. *PLoS One* **10**: e0136314.

Zhang J, Lee D, Dhiman V, Jiang P, Xu J, McGillivray P, Yang H, Liu J, Meyerson W, Clarke D. 2020. An integrative ENCODE resource for cancer genomics. *Nature communications* **11**: 1-11.
